# [Retracted] Icariin inhibits oral squamous cell carcinoma cell proliferation and induces apoptosis via inhibiting the NF-κB and PI3K/AKT pathways

**DOI:** 10.3892/etm.2026.13131

**Published:** 2026-03-16

**Authors:** Ling Sun, Jing Zhang

Exp Ther Med 22:942, 2021; DOI: 10.3892/etm.2021.10374

Following the publication of this paper, it was drawn to the Editor's attention by a concerned reader that the flow cytometric assay data shown for the ‘5 μM Icariin’ experiment in Fig. 2A on p. 4 was almost identical to a flow cytometric plot featured in another article written by different authors at different research institutes (albeit the city was the same) that had already been submitted for publication to the journal *Experimental and Therapeutic Medicine*.

Owing to the fact that the contentious data in the above article was strikingly similar to data that had already been submitted for publication, the Editor of *Experimental and Therapeutic Medicine* has decided that this paper should be retracted from the Journal. The authors were asked for an explanation to account for these concerns, but the Editorial Office did not receive a reply. The Editor apologizes to the readership for any inconvenience caused.

